# Changes in Brain Tissue and Behavior Patterns Induced by Single Short-Term Fasting in Mice

**DOI:** 10.1371/journal.pone.0080085

**Published:** 2013-11-05

**Authors:** Yuko Hisatomi, Kyo Asakura, Kenji Kugino, Mamoru Kurokawa, Tomiko Asakura, Keiko Nakata

**Affiliations:** 1 Department of Health and Nutrition Science, Faculty of Health and Social Welfare Science, Nishikyushu University, Kanzaki, Saga, Japan; 2 Dietary Department, Special Nursing Home for the Aged, Social Welfare Juridical Person Shiun-kai, Bungo-ono, Ooita, Japan; 3 Division of Nutritional Science, Graduate School of Human Health Science, University of Nagasaki, Nagasaki, Japan; 4 Department of Medical and Dental Sciences, Graduate School of Biomedical Sciences, Nagasaki University, Nagasaki, Japan; 5 Department of Applied Biological Chemistry, Graduate School of Agricultural and Life Sciences, The University of Tokyo, Bunkyo, Tokyo, Japan; Tokai University, Japan

## Abstract

In humans, emaciation from long-term dietary deficiencies, such as anorexia, reportedly increases physical activity and brain atrophy. However, the effects of single short-term fasting on brain tissue or behavioral activity patterns remain unclear. To clarify the impact of malnutrition on brain function, we conducted a single short-term fasting study as an anorexia model using male adult mice and determined if changes occurred in migratory behavior as an expression of brain function and in brain tissue structure. Sixteen-week-old C57BL/6J male mice were divided into either the fasted group or the control group. Experiments were conducted in a fixed indoor environment. We examined the effects of fasting on the number of nerve cells, structural changes in the myelin and axon density, and brain atrophy. For behavior observation, the amount of food and water consumed, ingestion time, and the pattern of movement were measured using a time-recording system. The fasted mice showed a significant increase in physical activity and their rhythm of movement was disturbed. Since the brain was in an abnormal state after fasting, mice that were normally active during the night became active regardless of day or night and performed strenuous exercise at a high frequency. The brain weight did not change by a fast, and brain atrophy was not observed. Although no textural change was apparent by fasting, the neuronal neogenesis in the subventricular zone and hippocampus was inhibited, causing disorder of the brain function. A clear association between the suppression of encephalic neuropoiesis and overactivity was not established. However, it is interesting that the results of this study suggest that single short-term fasting has an effect on encephalic neuropoiesis.

## Introduction

The number of patients with eating disorders in Japan has increased 10-fold since 1980 [1]. Other countries such as Europe and the United States are also prone to eating disorders. Psychological factors, home environment, and genetic factors are involved in the development of eating disorders in a complex manner. In addition, as indicated by the increase in the number of patients with eating disorders in our country in recent years, young people are strongly influenced to become slim. The National Health and Nutrition Survey in Japan has shown that approximately 20% of the lean population (body mass index [BMI] < 18.5 kg/m^2^; BMI = body mass [kg] / stature [m^2^]) are in their 20s. Many people who have a desire to lose weight quickly by extreme dietary restrictions, such as low-energy food intake and skipping meals, often lose more weight than necessary.

 The main eating disorders are bulimia nervosa and anorexia nervosa. Anorexia nervosa is the fear of gaining weight and a strong desire to be slim. It develops by excessive weight reduction owing to dietary restrictions and repeated episodes of fasting. According to a survey of junior high school students, conducted by the Ministry of Health, Labour and Welfare (2011), 2 out of every 100 students have an eating disorder, would have a few times including the reserve forces, young people of the age of onset is serious. Severe eating disorders manifest as bodily abnormalities such as atrophy of the brain and other organs, osteoporosis, and amenorrhea, and behavioral abnormalities such as overactivity despite malnutrition. Using brain CT images, studies have confirmed atrophy of the cerebral cortex and enlargement of the ventricles in anorexic patients [2,3]. The rate of weight loss is correlated to the degree of brain atrophy; however, the size of the brain can return to normal once body weight is restored [4]. In contrast, the effect of single short-term fasting on brain function is unknown. Whether short-term fasting causes atrophy and functional decline of the central nervous tissue has not been clarified thus far.

To clarify the impact of malnutrition on brain function, we conducted a single short-term fasting study as an anorexia model using male adult mice and determined if changes occurred in migratory behavior as an expression of brain function and in brain tissue structure.

## Materials and Methods

**Figure 1:**
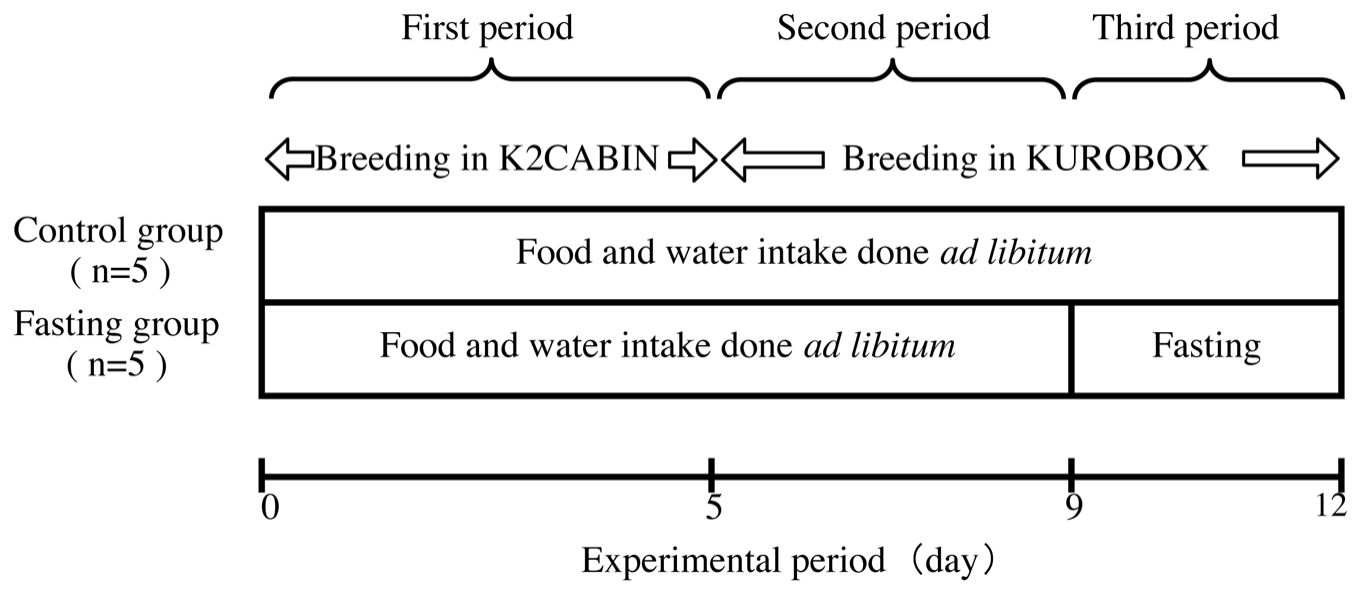
Experimental design. The mice were given CE-2 (CLEA Rodent Diet CE-2 for breeding) standard pellet chow. Tap water was used for drinking and was given ad libitum. Before the start of the experiment, mice were fed powder chow during a week-long acclimatization period in order to measure the feed intake in normal mice. In the first period, we measured food and water intake, and intake time using K2CABIN (Figure 2). In the second period, we measured the amount and rhythm of movement behavior using KUROBOX (Figure 2). In the third period, mice were fasted for 3 days, only drinking water was given ad libitum.

The experimental design is shown in Figure 1. C57BL/6J male mice (16 weeks of age, obtained from CLEA Japan Co. Ltd.) were equally divided into a control group and a fasted group (n = 5, each). The mice were housed in the K2CABIN (Phenotype Analyzing Co. Ltd, Japan) for the first experimental period and in the KUROBOX (PhenotypeAnalyzing Co. Ltd, Japan) for the second and third periods. The animal experiment was performed in the summer of August. The mice had access to tap water *ad libitum* and were fed a standard diet (CLEA Rodent Diet CE-2). The compositions of the experimental diets are shown in Table 1. The mice were fed powdered chow for the first period in the K2CABIN, and pelleted chow, containing the same ingredients, was fed for the second and third periods. During the first and second periods, mice received *ad libitum* access to water and food. During the third period, the fasted group had no access to food for 3 days but was allowed free access to water. The animal holding room was soundproofed, temperature controlled, (21 ± 2°C), and under a constant light-dark cycle (12:12 hr) with lights on at 7:00 am. 

**Table 1:** Composition of experimental diets (g/100g).

Ingredient	Amount
Moisture	8.9
Crude protein	24.9
Crude fat	4.6
Crude fiber	4.1
Crude ash	6.6
NFE	51.0
Total energy (Kcal/100g)	345

**Figure 2:**
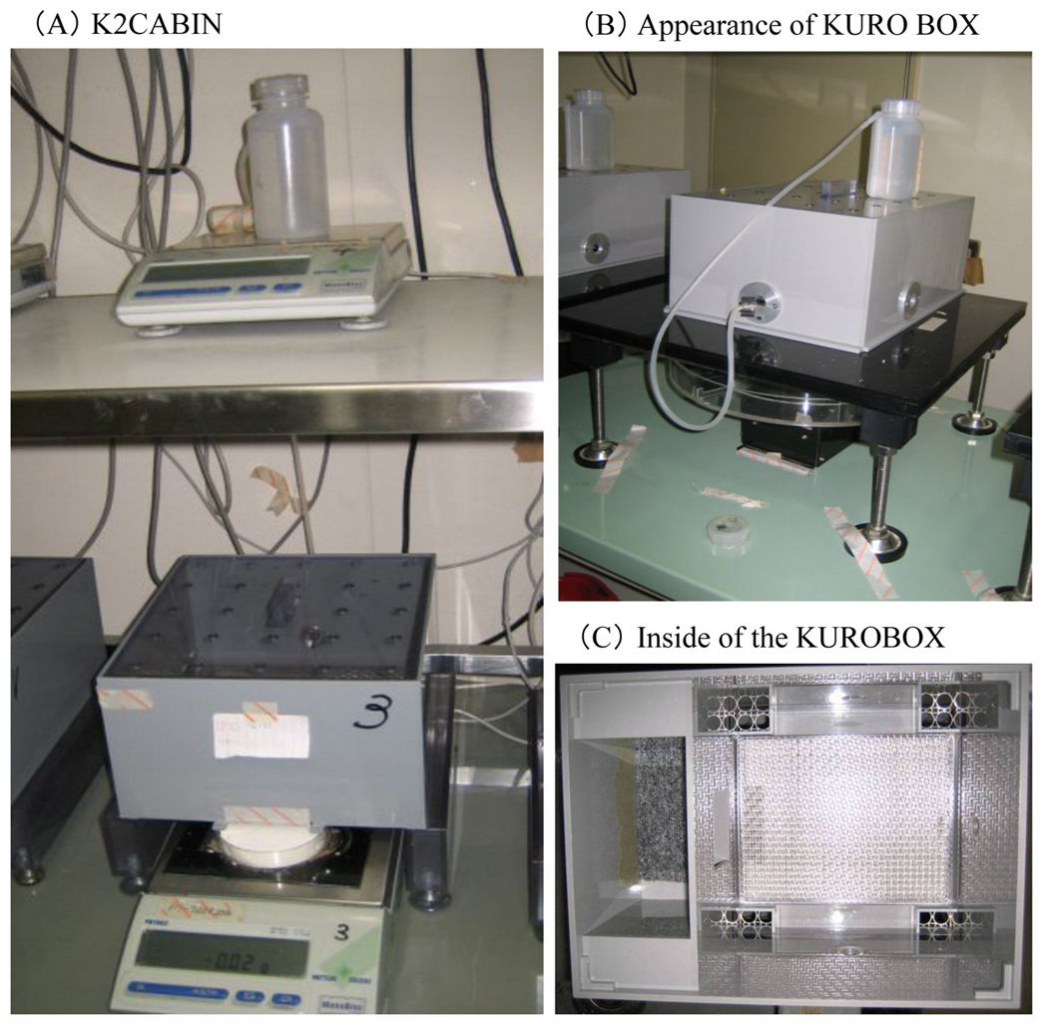
K2CABIN system and KUROBOX system. (A) K2CABIN system used to measure eating patterns and KUROBOX system used to measure movement patterns of behavior. (B),(C) KUROBOX; This measures the movement of mice by infrared sensors. We calculated the distance travelled by the mouse, the speed and the movement angle over time.

The drinking and eating behavior patterns of mice were measured using the K2CABIN system [5,6], which was developed by the authors (Figure 2-A). The K2CABIN system was used as an isolated breeding enclosure for studying the feeding behavior of resident mice. A well-shaped food hole was installed in the corner of the enclosure. A food tray, containing powdered feed was placed on the weighing instrument. Since mice cannot carry the powdered food, they consumed the food directly from the food tray. The amount of food ingested was accurately measured by the weighing instrument, which is a system that automatically records the weight 10 times within a 3-minute interval and averages the values ​​continuously. We analyzed the rhythm and eating pattern from these measurements. Running distance and movement patterns of the mice were measured using the KUROBOX system [7,8], which was developed by the authors (Figure 2-B, C). The KUROBOX system is an isolated breeding enclosure and a behavioral measurement instrument for studying the rearing of resident mice. The KUROBOX can calculate running distance and speed, and the direction of movement by measuring the center-of-gravity position of the movement trajectory by an infrared sensor. The temporal change of each movement can therefore be analyzed [7,8].

 In order to preserve the microstructure of the brain tissue, we immediately performed a thoracotomy after cervical dislocation of the mice. The mice were perfused, transcardially, with 50 mL of phosphate buffer into the left ventricle followed by 30 mL of 5% paraformaldehyde, and immediately decapitated. Brain tissue was excised from the dorsal side of the skull and wet weight was recorded with the blood vessels and brain membrane attached. In order to observe the brain tissue microscopically, the tissues were sectioned and visualized using the Kluver-Barrera (KB) staining [9], and immunostained [9,10] with the nestin antibody, which is a nerve trunk cell marker. We observed the micro indentations surrounding the cerebral ventricle and hippocampal dentate gyrus.

 A Student’s t-test was used for all statistical analysis. This study was approved by the Animal Use Committee of University of Nagasaki (approval number, 23-28) and was carried out as per the guidelines for animal experiments of University of Nagasaki and Law No. 105 and Notification No. 6 of the Government of Japan.

## Results

**Figure 3:**
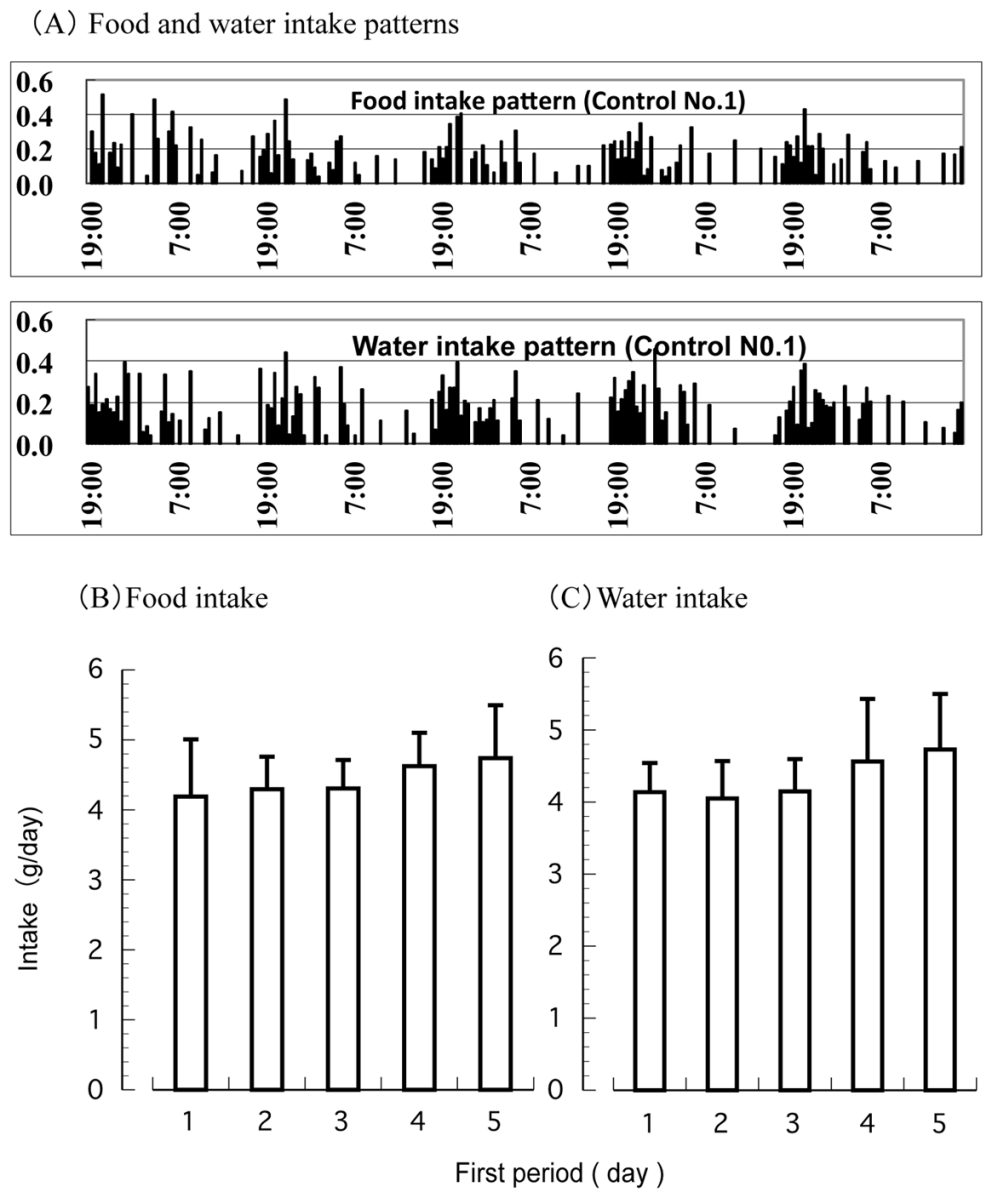
Food and water intake of mice in the first period under ad libitum. (A) An example of the food and water intake pattern in a mouse. (B) Average food intake. Values are Mean ± S.D. (n = 10). (C) Average water intake. Values are Mean ± S.D. (n = 10).

 The mice were nocturnal and hyperactive during the dark period. During the first experimental period, food and water were provided *ad libitum*, and the chronological food and water intake of every mouse were measured by the K2CABIN. Active responses for water and diet consumption were observed for the dark period (19:00–07:00). An example of the food and water intake patterns of the mice are shown in Figure 3-A. A similar pattern was found in all individuals of the 2 groups. The amount of food and water consumed did not change drastically throughout the 5 days of the first period (Figures 3-B, 3-C).

**Figure 4:**
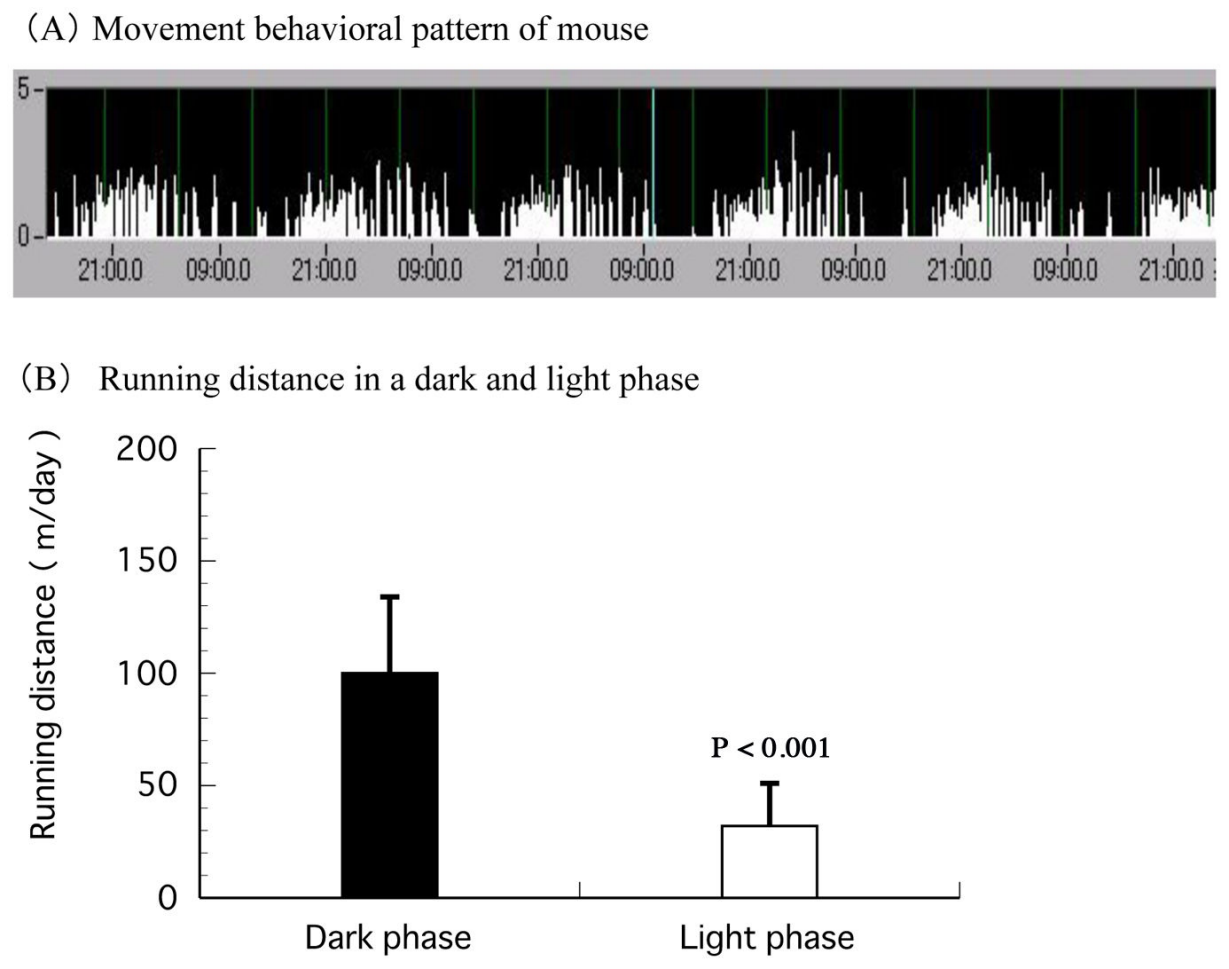
Movement behavioral patterns and running distance of mice in the second period given ad libitum intake of food and water. (A) An example of the movement behavioral pattern of a mouse in the second period. (B) Running distance in a dark and light phase in the second period. Values are Mean ± S.D. (n = 10). P < 0.001, significantly different from the dark phase value.

**Figure 5:**
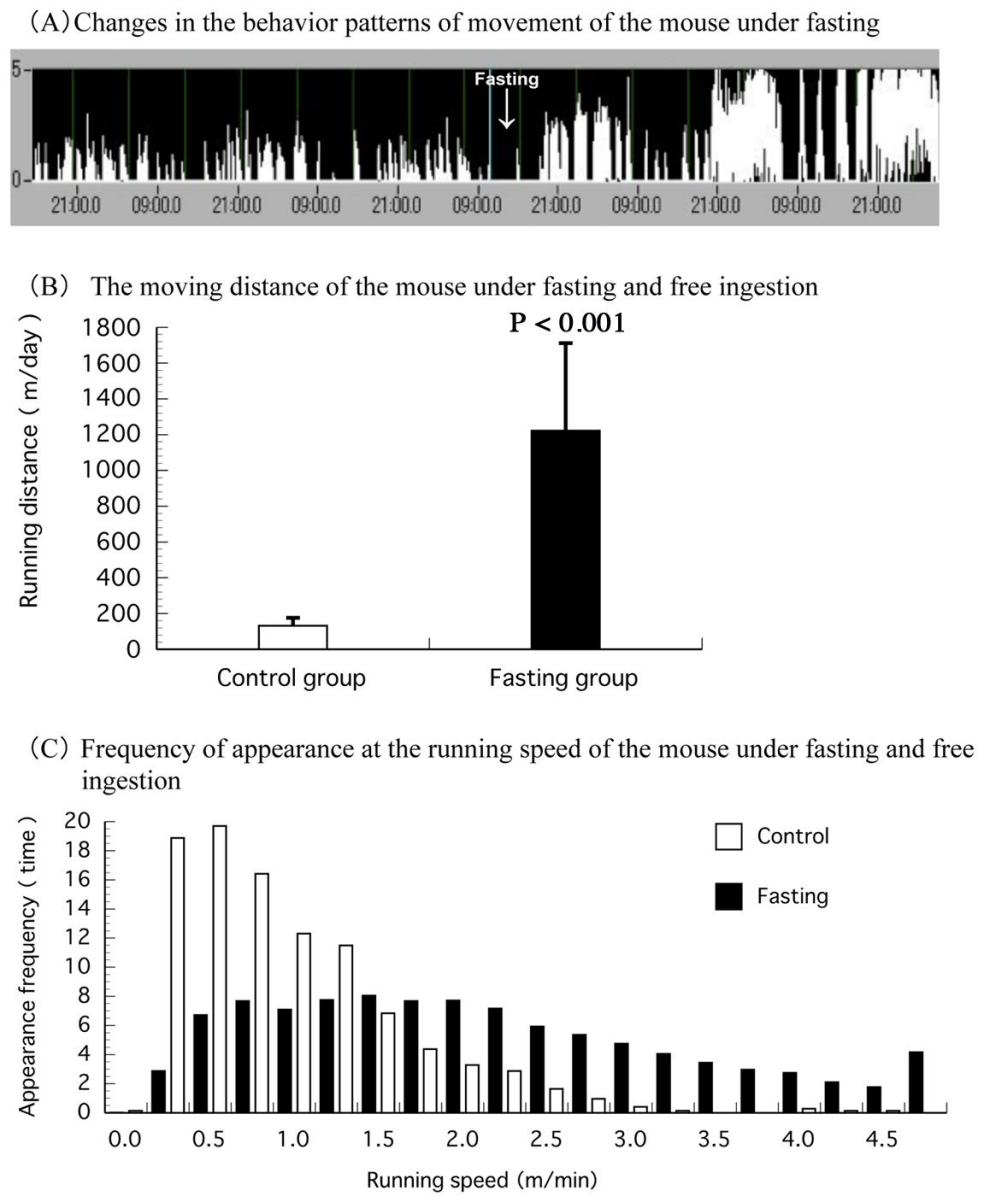
Movement behavioral patterns, running distance and frequency of appearance at the running speed of mice in the third period. (A) shows the behavior movement of a mouse under fasting in the third period. (B) shows the movement distance of the mice in the control (□) and fasting group (■). Values are Mean ± S.D. (n = 5). P < 0.001, significantly different from the control value. Compared to the control group, we observed that moving distance had increased significantly, and activities were enhanced in the fasting group. (C) the frequency of appearance at the running speed in control (□) and fasting (■). We compared the frequency distribution of each movement speed in each group. The interval of the X-axis is every 0.25 m. We observed that movement increased significantly and there was over-activity during fasting.

 The mice showed a typical pattern for nocturnal movement; they were inactive during the light phase and active during the dark phase (Figure 4-A). A similar pattern was found in all individuals of the 2 groups. The mice ran a distance of approximately 100 m/12 hours during the dark phase and 30 m/12 hours during the light phase (Figure 4-B). For the fasted group, the movement pattern increased in activity and became more pronounced over the second and third day of fasting. (Figure 5-A). Running distance significantly increased to approximately 1200 m/day in the fasted group compared to 130 m/day in the control group (Figure 5-B). 

 In addition, significant changes occurred in the movement pattern of the fasted mice (Figure 5-C) as the frequency distribution for each speed, from low to high-speed, revealed quicker movements for the fasted mice when compared to the control group. Excessive activity was observed in the fasted mice (Figure 5-C). 

 The body weight of the mice was reduced to approximately 70% of the initial weight after 3 days of fasting. In the macroscopic findings at autopsy, atrophy was observed in the tissues of the body, but not in the brain. There were no changes in the wet weight of the brain between groups. 

**Figure 6:**
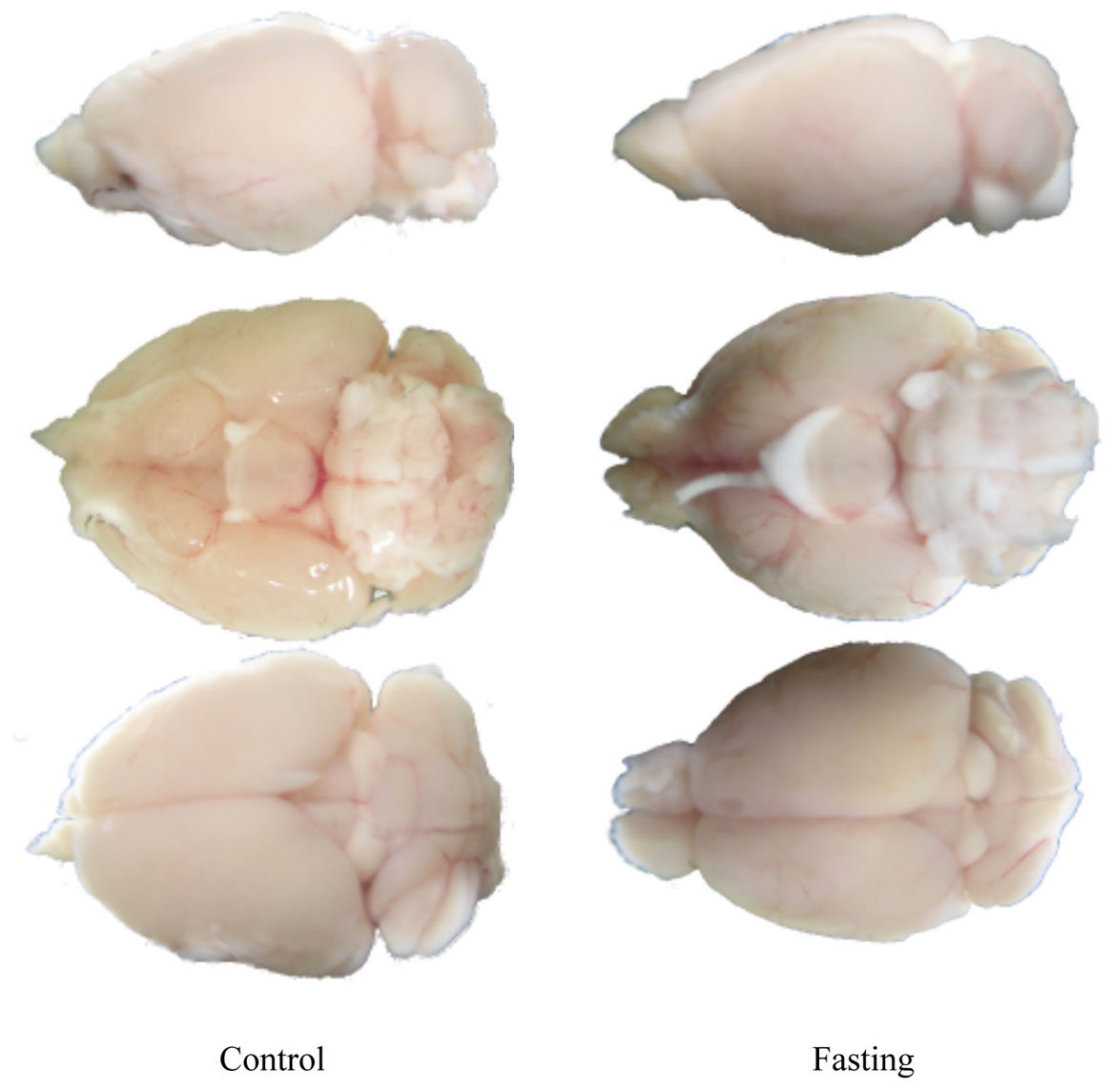
Macroscopic images of brain tissue. Macroscopically, upon dissection, we could not see any atrophy upon fasting and there was no change in wet weight of the brain. However, the surface of brain tissue appeared less glossy and seemed to have lost precision.

 However, it was observed that the tissue gloss on the surface of the brain was duller and the anatomical structures of the brain were less defined in the fasted mice (Figure 6).

**Figure 7:**
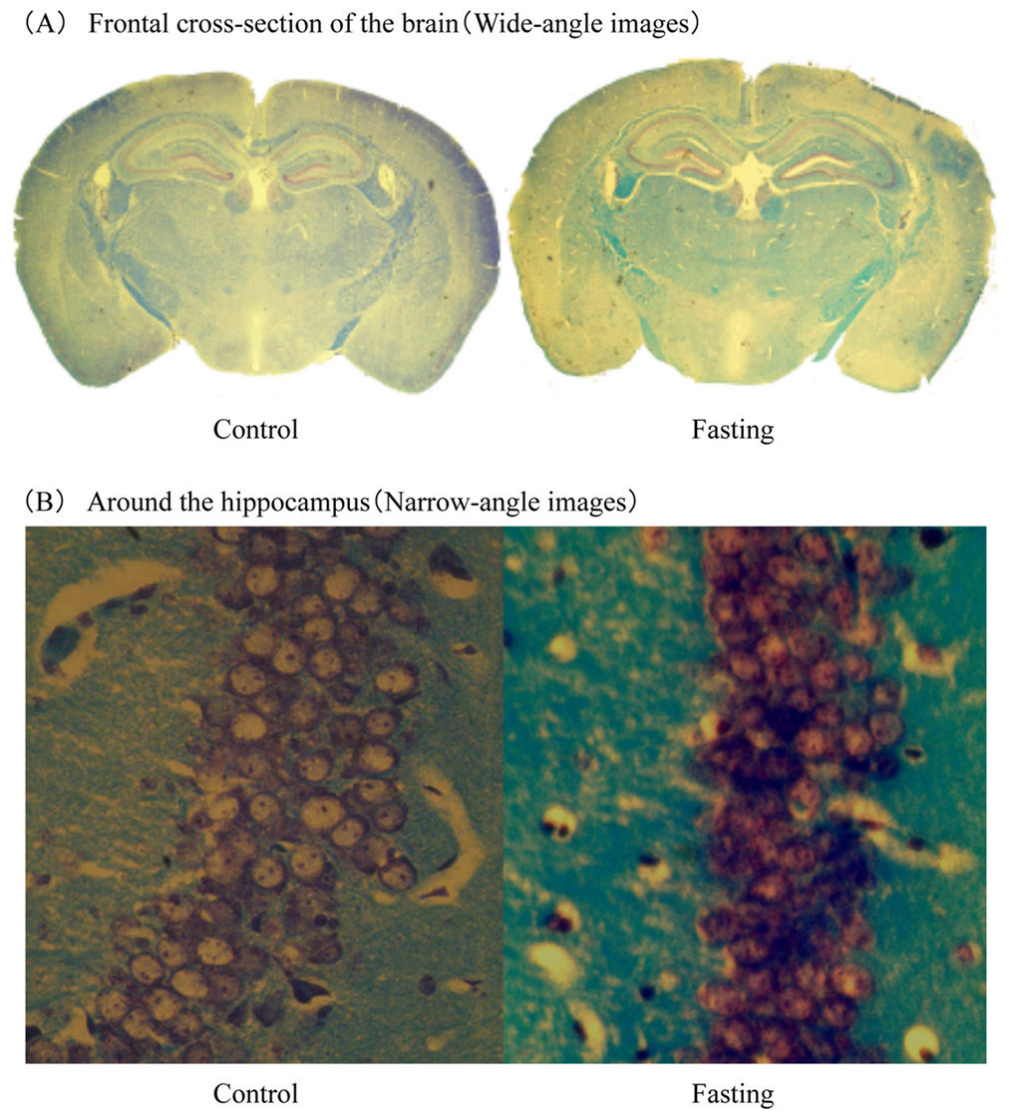
Optical microscopy images of brain tissue. Kluver–Barrera stained images of Frontal cross section of the brain (A) and the hippocampal region (B).

 In optical microscopic observation of the brain tissue by KB staining, we were not able to find a clear structural difference between fasted and control groups. (Figure 7-A, Figure 7-B). 

**Figure 8:**
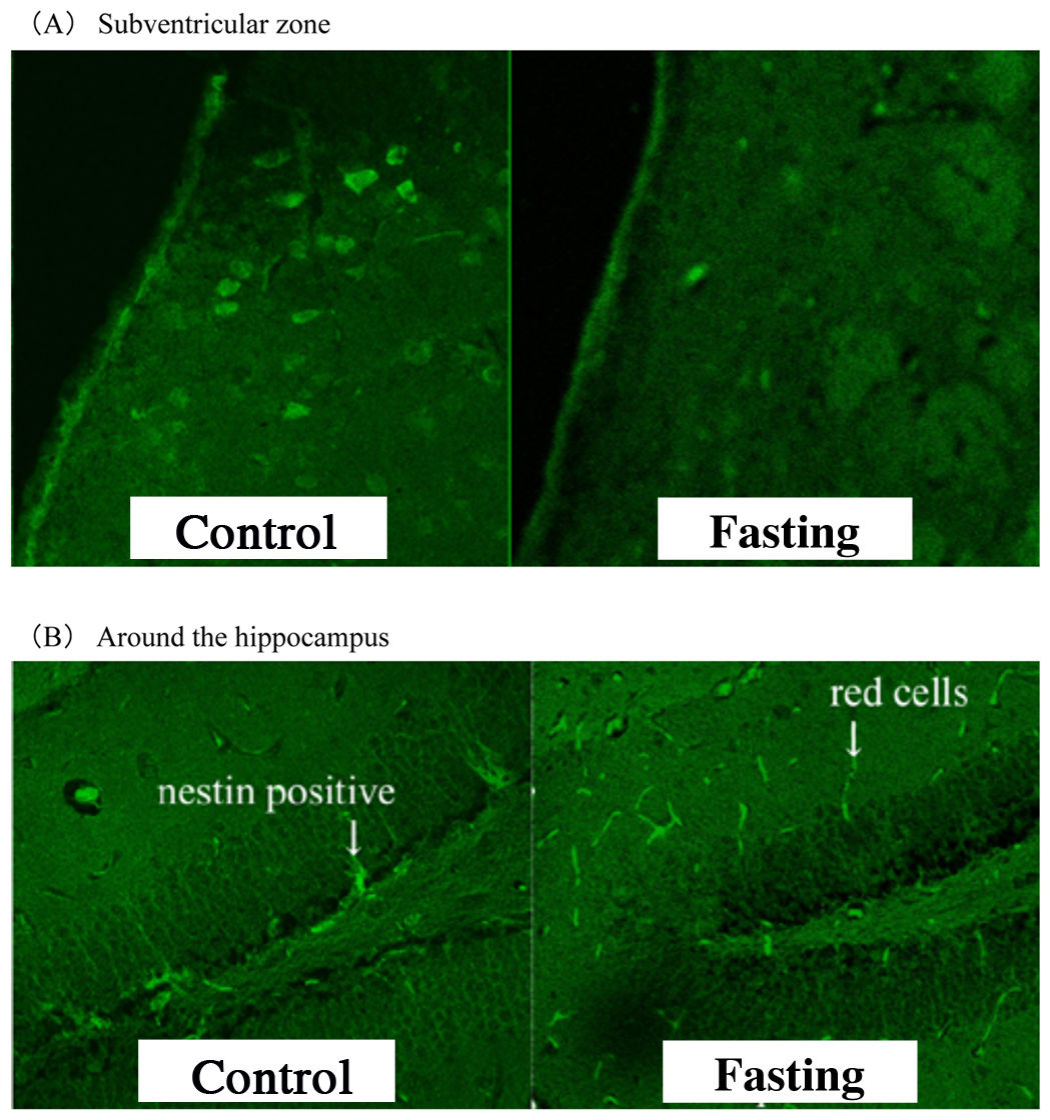
Fluorescent treponemal antibody imaging of the brain tissue. Brain sections of the subventricular zone (A) and hippocampus (B) were stained with immunostaining using nestin antibody. We observed the disappearance of nestin-positive cells in the fasting group.

 However, microscopic observation of brain tissue by immunostaining, showed a disappearance of nestin-positive cells in the hippocampal dentate gyrus and subventricular zone (SVZ) (Figures 8-A, 8-B) in the fasted mice. This suggests that neurogenesis was regressed by the 3 days of fasting. This finding is of great consequence because it appears that physiological brain tissue damage is caused by fasting.

## Discussion

To investigate the effect of single short-term fasting on the structure and function of the brain, we observed migratory behavior patterns during fasting and changes in brain tissue after fasting in adult male C57BL/6J mice.

Mice are nocturnal in that they are inactive during the day and hyperactive during the night. However, it was found that the rhythm of the activity of the mice changed after fasting. During fasting, the momentum and agility of the mice significantly increased. In this experiment, we found that fasting did not only increase the momentum of the mouse but also enhanced stereotyped movements. In experiments with rats, it was reported that insufficient intake corresponded to an increase in the arousal level of the brain and in the amount of locomotor activity [11]. Even in humans, overactivity is a phenomenon that is often observed in anorexia. The hypothalamus controls the body’s biological rhythms and neural pathways (present in various hypothalamic nuclei), and the biological rhythms of the body are sensitive to light and feeding stimulants. In mice, abnormal function may possibly originate in the hypothalamus [5].

From the results of the present study and past research results [11-17], it became clear that fasting affects cerebral activity. It is inferred that significant malnutrition (nutritional deficiency) affects the brain tissue structure, as confirmed by the imaging studies that demonstrated lesions in the brain. Cerebral atrophy is often found in patients with anorexia [2,3]. In a study examining the cerebral white matter lesions in dialysis patients, the possibility of a higher score for white matter lesions corresponded to the number of indicators for malnutrition [18]. 

In the 3 days of single fasting in this experiment, atrophy of the brain tissue was not observed. However, since the brain surface was not as glossy or as well defined, it is possible that some changes may occur in glycochain or phospholipid configuration in the cerebral cortex surface during fasting.

In this model animal experiment, cerebral atrophy was not found. Therefore, given that fasting in patients with anorexia occurs on a regular basis, cerebral atrophy may develop gradually in these patients.

Light microscopic examination of the brain tissue using KB stain did not reveal a distinct change after fasting. However, after immunostaining, nestin-positive cells were observed in the SVZ and the dentate gyrus of the hippocampus in the control group, but not in the fasted group. 

The nestin antibody is a neural stem cell marker that attaches to cells in all stages of neuron differentiation [19,20]. In this study, the nestin-positive cells were largely visible in the tissue surrounding the hippocampus, indicating neurogenesis in the control mice. However, nestin-positive cells were absent in the fasted group, suggesting that neurogenesis is inhibited in the dentate gyrus of the hippocampus and the SVZ. 

Current knowledge about the effects of fasting on the brain is poor. On the topic of nutrition, determining the relationship between short-term fasting and brain function is of particular interest and should be addressed in further investigations. A clear association between the suppression of encephalic neuropoiesis and overactivity was not established in the present study. However, it is interesting that the results of this study suggest that single short-term fasting has an effect on encephalic neuropoiesis.
